# Surface Functionalization by Stimuli-Sensitive Microgels for Effective Enzyme Uptake and Rational Design of Biosensor Setups

**DOI:** 10.3390/polym10070791

**Published:** 2018-07-19

**Authors:** Larisa V. Sigolaeva, Dmitry V. Pergushov, Marina Oelmann, Simona Schwarz, Monia Brugnoni, Ilya N. Kurochkin, Felix A. Plamper, Andreas Fery, Walter Richtering

**Affiliations:** 1 Department of Chemistry, M.V. Lomonosov Moscow State University, Leninskie Gory 1/3, 119991 Moscow, Russia; pergush@belozersky.msu.ru (D.V.P); ikur@genebee.msu.ru (I.N.K.); 2 Leibniz-Institut für Polymerforschung Dresden e.V., Hohe Str. 6, 01069 Dresden, Germany; oelmann@ipfdd.de (M.O.); simsch@ipfdd.de (S.S.); fery@ipfdd.de (A.F.); 3 Institute of Physical Chemistry II, RWTH Aachen University, Landoltweg 2, 52056 Aachen, Germany; brugnoni@pc.rwth-aachen.de (M.B.); plamper@pc.rwth-aachen.de (F.A.P.); richtering@rwth-aachen.de (W.R.); 4 N.M. Emanuel Institute of Biochemical Physics, Russian Academy of Sciences, Kosygina Str. 4, 119334 Moscow, Russia; 5 Physical Chemistry of Polymeric Materials, Technical University of Dresden, Hohe Str. 6, 01069 Dresden, Germany

**Keywords:** microgel, stimuli-sensitivity, surface modification, adsorption, streaming potential, quartz crystal microbalance with dissipation monitoring, biosensor, choline oxidase, poly(*N*-isopropylacrylamide-*co*-*N*-(3-aminopropyl)methacrylamide), poly(*N*-isopropylacrylamide-*co*-*N*-[3-(dimethylamino)propyl]methacrylamide)

## Abstract

We highlight microgel/enzyme thin films that were deposited onto solid interfaces via two sequential steps, the adsorption of temperature- and pH-sensitive microgels, followed by their complexation with the enzyme choline oxidase, ChO. Two kinds of functional (ionic) microgels were compared in this work in regard to their adsorptive behavior and interaction with ChO, that is, poly(*N*-isopropylacrylamide-*co*-*N*-(3-aminopropyl)methacrylamide), P(NIPAM-*co*-APMA), bearing primary amino groups, and poly(*N*-isopropylacrylamide-*co*-*N*-[3-(dimethylamino) propyl]methacrylamide), P(NIPAM-*co*-DMAPMA), bearing tertiary amino groups. The stimuli-sensitive properties of the microgels in the solution were characterized by potentiometric titration, dynamic light scattering (DLS), and laser microelectrophoresis. The peculiarities of the adsorptive behavior of both the microgels and the specific character of their interaction with ChO were revealed by a combination of surface characterization techniques. The surface charge was characterized by electrokinetic analysis (EKA) for the initial graphite surface and the same one after the subsequent deposition of the microgels and the enzyme under different adsorption regimes. The masses of wet microgel and microgel/enzyme films were determined by quartz crystal microbalance with dissipation monitoring (QCM-D) upon the subsequent deposition of the components under the same adsorption conditions, on a surface of gold-coated quartz crystals. Finally, the enzymatic responses of the microgel/enzyme films deposited on graphite electrodes to choline were tested amperometrically. The presence of functional primary amino groups in the P(NIPAM-*co*-APMA) microgel enables a covalent enzyme-to-microgel coupling via glutar aldehyde cross-linking, thereby resulting in a considerable improvement of the biosensor operational stability.

## 1. Introduction

Surface modification by polymers is a rapidly developing field of materials science, which attracts ever-increasing attention from researchers nowadays because of its many applications. It enables not only a significant change in surface properties, such as wettability, adhesion, and roughness, but also the introduction of new functionalities to the interface. As important examples of surface modification by polymers, grafting-from approach [1], layer-by-layer technique [2,3], electrodeposition [4], and so on, are to be mentioned.

Recently, we have shown that the surface modification of conductive substrates by functional polymers via their simple adsorption under the right conditions allows for the subsequent efficient binding of biomolecules, where the polymer layer acts as peculiar ‘glue’ keeping such bioactive species firmly at the interface [5,6]. With the help of this approach, easily-preparable enzymatic biosensor setups were constructed and applied for electrochemical detection of choline [5,6,7,8], phenol [5,9,10], blood esterases [9,11], and organophosphates [12].

Among the polymers we used for the surface modification, temperature-sensitive microgels, in particular, those based on poly(*N*-isopropylacrylamide) (PNIPAM), are of especial interest [13,14]. An advantageous feature of such microgels is the ease of their synthesis, with broad possibilities to control the microgel’s characteristics, such as mesh size, particle size, charge, and so on, complemented by facile functionalization [15]. The latter often comprises the incorporation of chargeable moieties into the microgels via the copolymerization of *N*-isopropylacrylamide with an ionic comonomer, such as, for example (meth)acrylic acid or (dialkyl)aminopropylmethacrylamide.

Because of the microgel’s soft nature, they tend to attach to solid interfaces in a pancake-like state [16,17,18,19], although the surface-bound microgels can then be strongly swollen. Furthermore, higher surface coverage can be reached by the microgels if their deposition takes place under conditions when they are in the collapsed state [10].

Electrostatic attraction can be used as a binding principle to load the PNIPAM-based ionic microgels with oppositely charged species, for example, small multivalent counterions [20,21], oppositely charged polyelectrolytes [22], proteins and enzymes [23,24], magnetic nanoparticles [25,26], or plasmonic nanostructures [27,28,29].

**Scheme 1:**
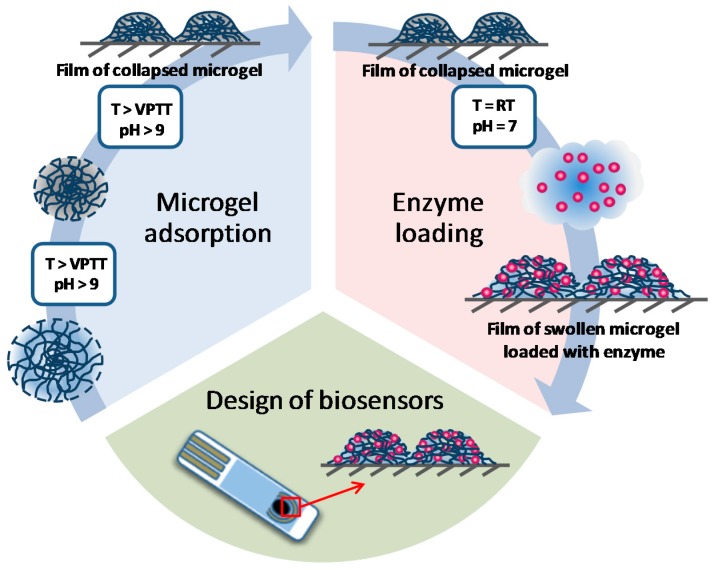
Bottom-up construction of microgel-based biosensor setups.

Previously, we found that such ionically functionalized microgels enable the capacious uptake of payloads [22]. Furthermore, they, being highly hydrated, appear to be favorable matrices for enzymes, perfectly preserving the catalytic activity of the biomolecules. It allowed us to successfully construct a number of biosensor systems, where different enzymes, that is, choline oxidase (ChO), tyrosinase (Tyr), and butyrylcholine esterase (BChE), were electrostatically bound to the cationic poly(*N*-isopropylacrylamide-*co*-*N*-[3-(dimethylamino)propyl]methacrylamide), P(NIPAM-*co*-DMAPMA), microgel [8,10,12]. We demonstrated that because of the delicate control of the pH and the temperature of adsorption of this stimuli-responsive microgel with cationic moieties, we can control its amount that is deposited onto graphite substrates, as well as the amount and localization of the enzymes subsequently taken up by the deposited microgel films. Remarkably, the loading of the microgel film with enzymes can further be enhanced if the conditions for their stimulated (‘spongelike’) uptake are met, that is, when the biomolecules are taken up under simultaneous swelling of the microgel particles [10]. Scheme 1 summarizes the bottom-up construction of the biosensor setups under the conditions of the stimulated adsorption of microgels (in uncharged state and at elevated temperature) and the subsequent enhanced loading of enzymes (binding under simultaneous swelling of collapsed microgels). Furthermore, a bienzymatic microgel-based biosensor system with spatially separated ChO and BChE was successfully fabricated, allowing for enzymatic cascade reactions to be realized [8].

The high swelling of microgels results in an open structure with highly mobile solvent and solute molecules in their particles along with rather high conformational mobility of microgel subchains. This can be advantageous, when the loaded microgels are intended to be used for controlled release applications. Alternatively, the same feature can become a drawback in biosensor technologies and can have adverse effects on the so-called operational stability, as biosensor constructs can gradually lose small enzyme molecules when such biosensors are applied for multiple measurements. This problem can be overcome if the cationic PNIPAM-based microgel contains primary amino groups, as one can covalently bind the enzyme globules to the microgel functional groups via cross-linking with glutar aldehyde (GA).

Aimed to enhance operational stability of the enzymatic microgel-based biosensor system, we synthesized poly(*N*-isopropylacrylamide-*co*-*N*-(3-aminopropyl)methacrylamide), P(NIPAM-*co*-APMA), microgel with primary amino groups. To understand further the processes of microgel/enzyme film formation and the role of the microgel’s stimuli-sensitivity at any step of the surface modification in more detail, we now examine and compare the deposition onto the conductive solid surfaces of the new P(NIPAM-*co*-APMA) microgel having primary amino groups, with the former one, P(NIPAM-*co*-DMAPMA), having tertiary amino groups. We also studied the subsequent interaction of an enzyme, ChO, with the films of the different microgels deposited onto such surfaces. Finally, the performance of the microgel/enzyme films as biosensors for choline was assessed. Furthermore, enzyme-to-microgel cross-linking with GA was examined as a powerful way to stabilize the resulting biosensor constructs.

## 2. Materials and Methods

ChO from *Alcaligenes sp.*, E.C. 1.1.3.17, activity 11.6 U/mg of protein, was purchased from Fluka (Germany), while its substrate, choline chloride, was received from Alfa Aesar (Karlsruhe, Germany). The buffers, 4-(2-hydroxyethyl)piperazine-1-ethanesulfonic acid; sodium salt (HEPES); tris(hydroxymethyl)aminomethane (TRIS), its hydrochloride (TRIS-HCl); and 3-(cyclohexylamino)-2-hydroxy-1-propanesulfonic acid (CAPSO) were obtained from Sigma-Aldrich (Steinheim, Germany). The initiator, 2,2′-azobis(2-methylpropionamidine) dihydrochloride (granular, 97%), was purchased from Sigma-Aldrich, and the cross-linker *N*,*N*′-methylenebis(acrylamide) and the surfactant *N*-cetyl-*N*,*N*,*N*-trimethylammonium bromide were received from AppliChem (Darmstadt, Germany). The comonomers *N*-isopropylacrylamide (NIPAM) and *N*-(3-aminopropyl)methacrylamide hydrochloride (APMA-HCl) were obtained from AcrosOrganics (Geel, Belgium) and Sigma-Aldrich, respectively. The samples of the P(NIPAM-*co*-APMA) microgel and the P(NIPAM-*co*-DMAPMA) microgel, the latter was used in this work for comparison, were synthesized by precipitation polymerization, according to the procedures reported by Gelissen et al. [30] and Mergel et al. [20], respectively. The manganese dioxide sol solution was prepared using potassium permanganate (Merck, Darmstadt, Germany) and manganese acetate tetrahydrate (AcrosOrganics). All of the other chemicals were of analytical grade and used without further purification. All of the aqueous solutions were prepared with deionized water (18.2 MΩ), purified by a Milli-Q water purification system (Billerica, MA, USA).

### 2.1. Laser Microelectrophoresis 

The electrophoretic mobility (EPM) measurements were performed at 25 °C on a NanoZS Zetasizer from Malvern (Malvern, UK) via the M3-PALS technique with a HeNe laser (λ = 633 nm) at the detection angle of 17°. The pH-dependence of the EPM was measured in disposable capillary cells (Malvern, DTS1061C), using a MPT-2 autotitrator from Malvern by titrating aqueous 0.1 g/L solutions of a microgel with 0.1 M NaOH. The mean of the three measurements at each pH-value is shown.

### 2.2. Potentiometric/Conductometric Titration

The potentiometric/conductometric titrations were carried out using a Methrohm (Filderstadt, Germany) 665 autotitrator in a thermostated titration cell at 25 °C. Next, 75.19 mg of the P(NIPAM-*co*-APMA) microgel was dissolved in 50 ml of Milli-Q water. Then, the microgel solution was transferred into the titration cell and its pH was adjusted to about 3 with 0.1 M HCl. After the equilibration of the solution for 15 min, the titration was performed by automatically adding portions of 2 μL 0.1 M NaOH. The conductivity and pH were measured simultaneously. The amount of the incorporated amine containing comonomer and the degree of protonation α of the microgel were calculated from the dependence of the pH on the volume of the added 0.1 M NaOH. The limiting values of α, that is, α = 0 and α = 1, were determined as the inflection points in the obtained conductivity curve.

### 2.3. Dynamic Light Scattering (DLS)

The DLS measurements were performed in pseudocross correlation mode with an ALV (Langen, Germany) setup comprising 633 nm HeNe laser (JDS Uniphase, Munich, Germany, 35 mW), a goniometer (ALV, CGS-8F), two avalanche photo diodes (Perkin Elmer, Wiesbaden, Germany, SPCM-CD2969), a digital hardware correlator (ALV 5000), and a light scattering electronics (ALV, LSE-5003). An external programmable thermostat Julabo F32 (Julabo, Seelbach, Germany) and an index-match-bath filled with toluene were used to provide the temperature control of the sample. The scattering angle was varied between 30° and 150°, in steps of 10°, and the measurement time was 60 s. The microgel samples were highly diluted to avoid any multiple scattering. The buffers that were used for preparations of samples were filtered three times through regenerated cellulose filters (Sartorius) with a pore size of 0.2 µm. The hydrodynamic radii, *R*_h,_ were calculated applying the Stokes-Einstein equation from *z*-average translational diffusion coefficients, derived from the second-order cumulant-fit analysis of the autocorrelation functions. The values of the polydispersity index were obtained from the second-order cumulant-fit analysis and were found to be less than 0.1 in all of the cases, thus indicating narrow size distributions for the P(NIPAM-*co*-APMA) and P(NIPAM-*co*-DMAPMA) microgels.

### 2.4. Electrokinetic Analysis (EKA)

The surface charge was examined using an Electrokinetic Analyzer (A. Paar KG, Graz, Austria). A pair of 34 mm × 25 mm samples with deposited microgel and microgel/enzyme films were used for each measurement. The values of the surface *ζ*-potential were calculated according to the Equation (1), as follows:

(1) $$\zeta =\frac{\eta }{\varepsilon_{0}\varepsilon_{r}}\frac{\Delta U}{\Delta p}\kappa ,$$

where Δ*U* is the streaming potential between a couple of Ag/AgCl electrodes located at the opposite ends of the samples; Δ*p* is the applied pressure (0–150 mbar); η, *κ*, and ε_r_ are the dynamic viscosity, conductivity, and relative dielectric permittivity of the flowing electrolyte solution (0.001 M KCl), respectively; and ε_0_ is the dielectric permittivity of the vacuum. In order to adjust pH of the flowing electrolyte solution in the range of 3–10, 0.1 M KOH or 0.1 M HCl were used, respectively.

### 2.5. Quartz Crystal Microbalance with Dissipation Monitoring (QCM-D)

The adsorption of the microgels and ChO was followed in situ by a QSense E1 system (Biolin Scientific, Gothenburg, Sweden) in a QSense flow-through cell. The sensor crystals (QSense) are gold-coated AT-cut quartz with gold-plated polished electrodes. Prior to the experiments, the crystals were cleaned, as described by Sigolaeva et al. [10]. Each QCM-D experiment started from the base line recording for the 10 mM CAPSO at pH 9.3 and 25 °C. If necessary, the temperature was set at 50 °C and the new base lines for the frequency and dissipation were recorded. Next, the microgel was adsorbed at a specified temperature (25 or 50 °C) from 1 g/L solution in 10 mM CAPSO at pH 9.3. A washing step with the same buffer solution was performed to remove any loosely attached material. Afterwards, the ChO was immediately allowed to interact with the adsorbed microgel film from a 0.4 g/L solution in 10 mM TRIS at pH 7.0 and 25 °C. To remove the weakly adsorbed enzyme globules, 10 mM TRIS of the same pH was used for rinsing. The frequency and dissipation shifts were continuously recorded as a function of time during each adsorption stage or temperature cycle. The QCM-D data were analyzed using the viscoelastic Voigt-based model of the integrated QTools software (Biolin Scientific, Gothenburg, Sweden). The viscoelastic microgel/ChO films were modeled as one layer with a fixed fluid density of 998 kg/m^3^, fixed layer density of 1020 kg/m^3^, and fixed fluid viscosity of 0.000893 kg/ms, and the modeled values for the layer viscosity, shear modulus, and mass of the layer were obtained. When needed (for adsorption experiments at 50 °C), the fluid viscosity was also switched from a fixed to modeled value, in order to obtain better fits.

### 2.6. Preparation of Surfaces

For the electrochemical experiments, the screen-printed electrodes (SPEs) were fabricated using a poly(vinyl chloride) (PVC) film with the thickness of 0.2 mm as a support. Conductive graphite paste (GwentGroup, Pontypool, UK) was applied via screen-printing through a 200 mesh screen stencil. Each SPE consisted of a round-shaped working area (2.5 mm diameter), a conductive track (30 mm × 1.5 mm), and a square extremity (3 mm × 7 mm) for electrical contact. For the EKA, 34 mm × 25 mm pieces of the same PVC films were covered by a layer of the same conductive graphite paste (GwentGroup, Pontypool, UK), deposited by a squeegee. All of the fabricated surfaces were premodified with a peroxide-sensitive layer of MnO_2_ nanoparticles. A sol of MnO_2_ nanoparticles was prepared via the mixing of equal volumes of 0.25 mM KMnO_4_ and 0.375 mM of Mn(CH_3_COO)_2_, followed by shaking the mixture for 5 min. To modify the surfaces of the SPEs, a 10 µL drop of the freshly prepared sol of MnO_2_ nanoparticles was applied on the working area of each electrode, followed by complete drying, rinsing with Milli-Q water, and heating at 60 °C for 1 h. The surfaces examined by EKA were premodified with a MnO_2_-layer via spin-coating of the above MnO_2_-sol solution. The MnO_2_-modified surfaces were stored dry at an ambient temperature until further use.

### 2.7. Microgel/Enzyme Film Assembling

The microgels were adsorbed onto a MnO_2_-premodified graphite surface at a specified temperature, in the range of 20–60 °C, via dip coating by dipping into the preheated 1 g/L dispersion of the microgel in 10 mM CAPSO/CAPS (pH 9.3/10.3) for 1 h. After that time had been elapsed, the surface was rinsed with Milli-Q water and gently dried. Then, the ChO was adsorbed in a similar way from the 4 g/L solution, in 10 mM TRIS of pH 7.0 at 25 °C, followed by rinsing with Milli-Q water and drying. The surfaces covered by microgels were stored at room temperature until further use. The surfaces covered by microgel/enzyme films were stored and refrigerated until further use.

### 2.8. Electrochemical Assay

The electrochemical amperometric measurements were performed with a micropotentiostate IPC-Micro (Kronas Ltd., Moscow, Russia), using a two-electrode configuration as described by Sigolaeva et al. [8]. The SPE modified by a microgel/enzyme film was used as the working electrode (active surface area of 0.049 cm^2^), while the Ag/AgCl (surface area of 1.03 cm^2^) served as a counter/reference electrode. The amperometric measurements were carried out in a 50 mM HEPES/30 mM KCl buffer (pH 7.5) at room temperature in a 1 ml one-compartment electrochemical cell under continuous stirring. The current generated in response to the addition of a choline solution at an applied potential of +450 mV vs. Ag/AgCl was recorded.

**Scheme 2:**
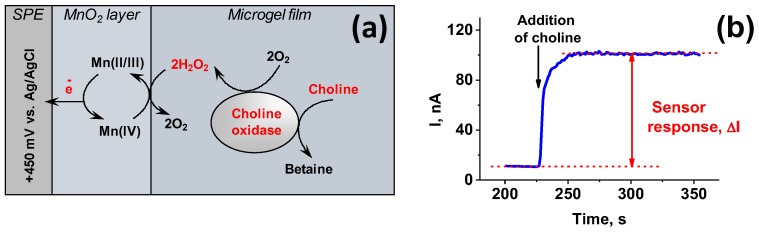
(**a**) Principle of electrochemical detection of choline and (**b**) a typical biosensor response to the addition of choline.

Scheme 2 represents the principle of the electrochemical detection of choline, along with a typical biosensor response. The sensor response of each SPE covered with a microgel/enzyme film was determined as the difference between an average value of the steady-state base line current before and after the addition of choline (Scheme 2). Each electrochemical result is presented as the mean ± standard deviation (SD) of three measurements obtained for at least three similarly prepared SPEs.

## 3. Results and Discussion

### 3.1. Stimuli-Dependent Behavior of Microgels in Aqueous Solutions

The microgel samples represent colloidally-stable dispersions of internally cross-linked polymer microparticles exhibiting dual-sensitivity to external stimuli (i.e., temperature and pH). In this study, we mainly addressed the examination of the P(NIPAM-*co*-APMA) microgel, which contains primary amino groups. It was synthesized in one step by the precipitation copolymerization of NIPAM (85 mol %) with APMA (10 mol %) as an ionic comonomer in the presence of cross-linking agent *N*,*N*′-methylenebis(acrylamide) (5 mol %). The second sample represents the P(NIPAM-*co*-DMAPMA) microgel with tertiary amino groups. This sample was synthesized according to the similar procedure with DMAPMA instead of APMA as an ionic comonomer (the actual amount of DMAPMA incorporated into the P(NIPAM-*co*-DMAPMA) microgel was about 9 mol %). All of the properties and characteristics of the P(NIPAM-*co*-DMAPMA) microgel from the new batch were similar to those of the one used before [10,20], with exception of the larger size of its particles. The characteristics of this microgel at different temperatures and pH-values are given in the Supplementary Materials (Supplementary Materials, Figures S1 and S2).

**Figure 1:**
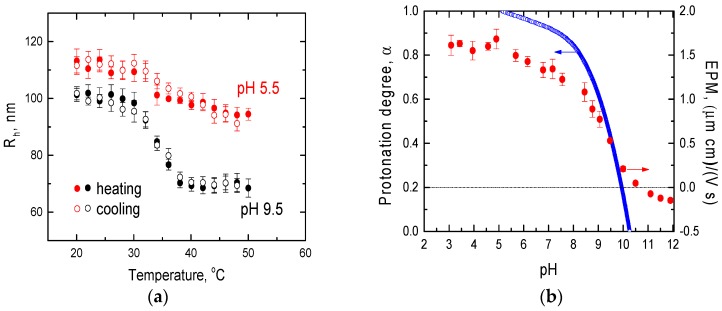
(**a**) The temperature dependence of the hydrodynamic radius of the P(NIPAM-*co*-APMA) microgel particles, where P(NIPAM-*co*-APMA) stands for poly(*N*-isopropylacrylamide-*co*-*N*-(3-aminopropyl)methacrylamide), at pH 5.5 (α ≅ 1.0) and pH 9.5 (α ≅ 0.45). (**b**) The pH-dependence of the protonation degree α of the P(NIPAM-*co*-APMA) microgel obtained from the potentiometric titration data (blue circles) and the electrophoretic mobility (EPM) values of the microgel particles at different pH-values obtained at 25 °C by laser microelectrophoresis (red circles).

The stimuli-dependent behavior of the P(NIPAM-*co*-APMA) microgel in an aqueous media is demonstrated by its sensitivity to variations of conditions, that is, to changes of temperature and of the pH, which was examined by DLS, potentiometry, and laser microelectrophoresis. The thermosensitivity of the microgel imparted by PNIPAM-subchains manifests itself as a reversible shrinking-swelling of its particles via cycling the temperature above and below the volume phase transition temperature (VPTT) (Figure 1a). The VPTT of the P(NIPAM-*co*-APMA) microgel was found to be around 33 °C, which is very close to that of the pure PNIPAM [31]. The temperature-induced change of the hydrodynamic size of the microgel particles ∆*R*_h_ (where *R*_h_ is the hydrodynamic radius) depends on the degree of the microgel protonation α, as its thermosensitivity is suppressed by the electrostatic repulsion of similarly charged groups of the ionic comonomer and the osmotic pressure of their counterions. The minimum ∆*R*_h_ of about 20 nm is observed at pH < 6.0, that is, in the pH-range corresponding to the fully (or nearly fully) protonated state of the P(NIPAM-*co*-APMA) microgel, as follows from the potentiometric titration data given in Figure 1b. With the decreasing protonation of the microgel, the ∆*R*_h_ value increases (e.g., ∆*R*_h_ is about 33 nm at a pH of 9.5, where only about a half of the ionic comonomer groups are protonated (Figure 1a)). However, the heating of the P(NIPAM-*co*-APMA) microgel in its fully deprotonated state (that is, at a pH of about 10.3) results in the formation of irreversibly precipitating aggregates (data not shown).

The pH-sensitivity of the microgel is imparted by the APMA comonomer units incorporated into its particles. The pH-dependence of the degree of microgel protonation (α vs. pH) shows a transition from the fully protonated (charged) state (α = 1), at a pH of about 5.2, to the fully deprotonated (non-charged) state (α = 0), at a pH of about 10.3 (Figure 1b). According to the potentiometric titration, the actual amount of APMA incorporated into the P(NIPAM-*co*-APMA) microgel is about 16 mol %. The corresponding EPM values of the microgel particles at different pH values are also shown in Figure 1b. This data is in line with the results of the potentiometric titration experiments, clearly manifesting the pH-sensitive behavior of the P(NIPAM-*co*-APMA) microgel. At pH ≤ 6.0, highly positively charged microgel particles exhibit the EPM values of about 1.5 (μm × cm)/(V × s), whereas the EPM decreases and reaches zero at a pH of about 10.5, thereby indicating their progressive deprotonation with the increasing the pH. A further increase in the pH induces a slight overcharging of the microgel particles (Figure 1b).

### 3.2. Microgel and Microgel/Enzyme Film Assembling on Solid Surfaces

Ultimately aimed at the design of electrochemical biosensor setups, we adsorb the P(NIPAM-*co*-APMA) microgel onto a conductive solid surface. Generally, the surface of our interest represents a thin layer of a conductive graphite paste, which is deposited onto a solid support through a screen-printing technique. This technique is rather simple and cheap, being widely distributed in biosensor technologies. In our specific case, a layer of conductive graphite paste was attached to a PVC film. This graphite-based surface is characterized by high roughness due to the presence of 3–10 μm graphite flakes, with the roughness within one flake being 86 ± 2 nm [6,12]. The high hydrophobicity of this surface results in a hard wettability, confirmed by the high value of the static contact angle of 133.3° ± 0.8°, to which a high roughness makes an additional contribution [5,6]. The initial graphite surface was additionally premodified with MnO_2_ nanoparticles to impart the surface sensitivity to hydrogen peroxide (see Experimental Part, and the Scheme 2 therein). Such a modification, however, does not result in any notable changes of the surface roughness and of the static contact angle [5,12]. The resultant surface is therefore very rough, hydrophobic, and hardly wetted by aqueous solutions.

**Figure 2:**
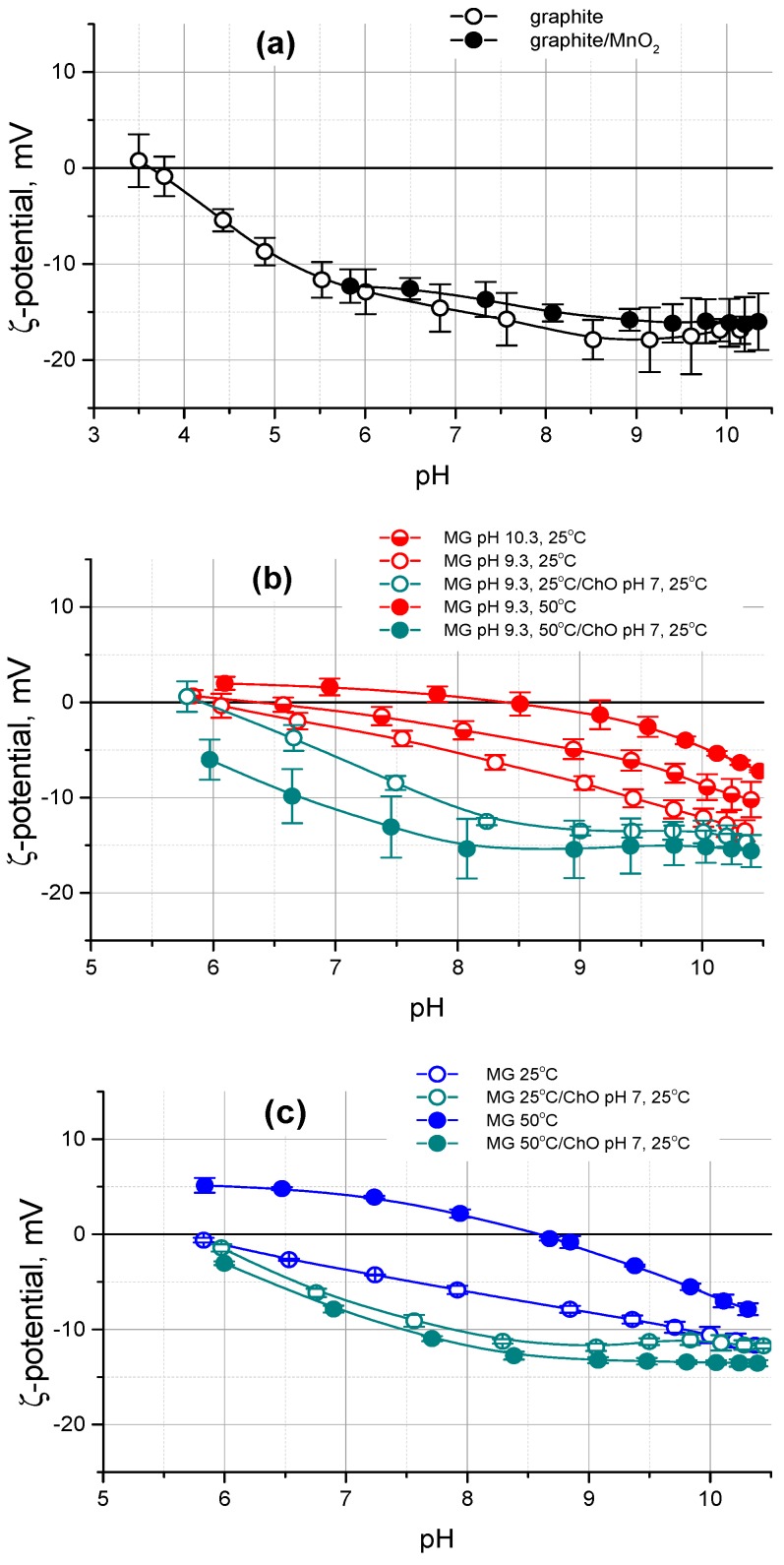
(**a**) *ζ*-potential as a function of the pH for the bare graphite surface and the graphite surface modified with MnO_2_; (**b**) *ζ*-potential as a function of the pH for the film of the P(NIPAM-*co*-APMA) microgel adsorbed at different pH-values and temperatures onto graphite surface premodified with MnO_2_, and further interacted with choline oxidase (ChO) at pH 7 and room temperature. (**c**) *ζ*-potential as a function of the pH for the film of the P(NIPAM-*co*-DMAPMA) microgel, where P(NIPAM-*co*-DMAPMA) stands for poly(*N*-isopropylacrylamide-*co*-*N*-[3-(dimethylamino)propyl] methacrylamide), adsorbed at pH 9.3 and different temperatures onto graphite surface premodified with MnO_2_ and further interacted with ChO at pH 7 and room temperature. The support is a poly(vinyl chloride) (PVC) film. MG is the abbreviation used for a microgel.

The surface *ζ*-potential, measured by EKA, is a sensitive measure of the changes of the surface charge. It provides information on the presence of negative or positive charges on the surfaces [32,33,34]. Applying this technique, the surface *ζ*-potentials of the bare PVC film, PVC film covered with a graphite layer, and the same one further premodified with MnO_2_ nanoparticles, were measured in the pH range of 3.5–10.5. While the bare PVC film is unexpectedly characterized by very negative surface *ζ*-potentials (Supplementary Materials, Figure S3), the modification with the graphite paste considerably shifts their values to less negative ones. Figure 2a shows that the bare graphite surface has the constant *ζ*-potential of (−15 ÷ −17) mV at a pH > 7, while it consequently becomes less negative below pH 7. The isoelectric point (IEP) of the surface is observed at the pH value of about 3.5. The graphite surface premodified with MnO_2_ nanoparticles shows a quite similar pH trend for *ζ*-potentials in the pH range of at least 5.5–10. As the MnO_2_ nanoparticles irreversibly lose their mediating properties toward hydrogen peroxide below pH 5.5 (for details see Sigolaeva et al. [5]), such measurements for the MnO_2_-modified graphite surface were not performed in the more acidic region (pH < 5.5), and hence the IEP for this surface was not determined either. However, one could reasonably assume that it is to be close to that of the bare graphite surface, because we have not seen, so far, any notable difference in the properties of the bare and MnO_2_-modified graphite surfaces.

The charge and hydrophobicity of the microgels controls the surface modification. With the help of EKA, we revealed the efficiency of this process for the P(NIPAM-*co*-APMA) microgel under different experimental conditions and compared it with that found for the P(NIPAM-*co*-DMAPMA) microgel applied for such purposes in our previous work [8,10,12]. For the effective adsorption of microgel particles onto graphite-based surfaces, we applied a strategy of stimuli-controlled modification, which we already described elsewhere [8,10,12] (see also Scheme 1). Following this strategy, one can expect the highest microgel adsorption in the pH range where all of the chargeable groups are deprotonated (α = 0), that is, at high pH values in the case of the P(NIPAM-*co*-APMA) and P(NIPAM-*co*-DMAPMA) microgels. To further facilitate the adsorption of the microgel particles onto the graphite-based surface, it is preferable to carry out their adsorption at elevated temperatures (*T* > VPPT), at which the microgels are in their deswollen and rather hydrophobic state.

Figure 2b shows that the negative *ζ*-potentials of the MnO_2_-modified graphite surface further covered with P(NIPAM-*co*-APMA) microgel substantially shift to higher (less negative or even positive) values over the whole pH-range examined in comparison with those of the initial surface. These drastic changes of the surface properties can be quantitatively characterized by the shift of the surface IEP. Both the increase of the pH or of the temperature of the dispersion from which the microgel particles are deposited leads to more efficient surface modification. This clearly manifests itself by the shift of the surface IEP from 3.5 (bare graphite surface) to 6.0, 6.5, or even 8.5 for the film of the P(NIPAM-*co*-APMA) microgel adsorbed at pH 9.3 (α = 0.5) and 25 °C, pH 10.3 (α = 0) and 25 °C, or pH 9.3 (α = 0.5) and 50 °C, respectively. As the IEP of the P(NIPAM-*co*-APMA) microgel in solution is about 10.5, one can infer that the MnO_2_-modified graphite surface is most probably not fully covered by microgel particles and the discontinuous polymer film is formed. Obviously, this leads to the lower values of the surface IEP, due to a contribution of the bare (uncovered) parts of the used surface.

These findings fit very well to our former results on the stimuli-controlled modification of graphite-based surfaces with the P(NIPAM-*co*-DMAPMA) microgel [8,10,12]. In the previous studies, however, the EKA was not applied to characterize the surface modification efficiency. For comparison purposes, we carried out similar experiments for the MnO_2_-modified graphite-based surfaces covered with this microgel, when it was deposited at pH 9.3 (α = 0) at two different temperatures of 25 and at 50 °C, which are below and above VPTT (VPPT = 40 °C), respectively. As follows from Figure 2c, an even a more pronounced shift of the values of the surface IEP, from about 5.7, when the P(NIPAM-*co*-DMAPMA) microgel was deposited at 25 °C, to about 8.5, when it was deposited at 50 °C, is observed in this case.

One can see that at pH 7, where the subsequent interaction of the enzyme with the adsorbed microgels is performed for the construction of the biosensor setups [8,10,12], the values of the surface *ζ*-potential shift from about −2.5 mV through 0 mV, to about +1.5 mV for the films of the P(NIPAM-*co*-APMA) microgel deposited at pH 9.3 (α = 0.5) and 25 °C, pH 10.3 (α = 0) and 25 °C, or pH 9.3 (α = 0.5) and 50 °C, respectively. An even higher surface overcharging is observed in the case of the P(NIPAM-*co*-DMAPMA) microgel adsorbed at pH 9.3 (α = 0). Indeed, the values of the surface *ζ*-potential change from about −3.5 mV when the microgel deposition was performed at 25 °C, to about +4 mV when it was carried out at 50 °C. As ChO, the enzyme used in this study, possesses the IEP of 4.8 [8], and therefore its globules at pH 7 bear net negative charge, the pH-dependences of the surface *ζ*-potential for the P(NIPAM-*co*-APMA) or P(NIPAM-*co*-DMAPMA) microgel films expectedly shift toward more negative (or less positive) values upon the further uptake of this enzyme by such microgel films, which was performed at pH 7 (Figure 2b,c).

Thus, the performed EKA allows for an understanding of how the conditions at which the microgel deposition takes place, influence the surface charge behavior of the resultant microgel films deposited onto MnO_2_-modified graphite-based surfaces, and specify the optimal conditions corresponding to the most efficient surface modification. Remarkably, the EKA appears to be a unique technique, which enables the evaluation of the surface modification efficiency for real microgel-based constructs*,* that is, microgel films deposited onto graphite-based layers on PVC supports, while the other techniques we applied so far, dealt with the microgel films formed on suitable model substrates, such as highly-oriented pyrolytic graphite (atomic force microscopy) and a gold layer on quartz resonator (QCM-D).

**Figure 3:**
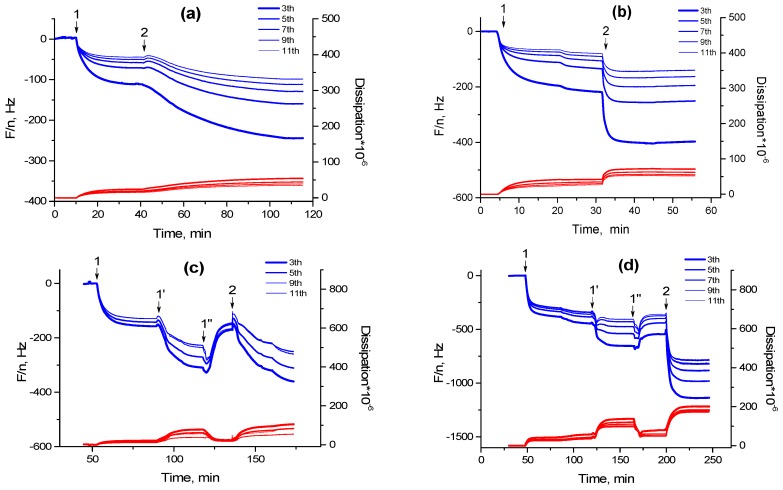
Normalized frequency, *F/n*, (blue lines) and dissipation, *D*, (red lines) shifts for gold-coated quartz crystal upon the adsorption of the P(NIPAM-*co*-APMA) microgel (**a**,**c**) or the P(NIPAM-*co*-DMAPMA) microgel (**b**,**d**), followed by the adsorption of ChO. Conditions: (Step 1) microgel adsorption from 1 g/L at pH 9.3 at 25 °C (**a**,**b**) or 50 °C (**c**,**d**); (Step 1′) temperature-induced swelling of the microgel film at pH 9.3 upon a temperature decrease from 50 to 25 °C; (Step 1″) temperature-induced deswelling of the microgel film at pH 9.3 upon a temperature increase from 25 to 50 °C; (Step 2) ChO uptake from the solution with the enzyme concentration of 0.4 g/L at pH 7.0 by the microgel film at 25 °C, in the case of the microgel film deposited at 50 °C, the enzyme uptake takes place simultaneously with microgel swelling upon a temperature jump from 50 to 25 °C (‘spongelike’ adsorption).

The latter technique (i.e., QCM-D) allows for following the adsorption of microgel particles and the uptake of enzyme globules in situ. The layer resonance frequency *f* and dissipation *D* at several overtones of the fundamental frequency resulting from QCM-D measurements are both sensitive to the mass of the adsorbed material and viscoelastic properties of the adsorbed film. Each QCM-D experiment was carried out in a flow-through cell and was started from base line recording for the 10 mM CAPSO at pH 9.3 and a specified temperature (25 or 50 °C). Then, the sample of each of the two microgels was first adsorbed at the specified temperature (25 or 50 °C) from a 1 g/L solution in 10 mM CAPSO at pH 9.3, followed by a washing step with the same buffer to remove any loosely attached material (Figure 3, step 1). In this way, we examined the adsorption of the P(NIPAM-*co*-APMA) and P(NIPAM-*co*-DMAPMA) microgels onto gold-coated quartz crystals at two different temperatures, at 25 °C, where both of the microgels are in the rather hydrophilic swollen state (Figure 3a,b), and at 50 °C, when both of the microgels are in their collapsed and rather hydrophobic state (Figure 3c,d). For the microgels adsorbed in their collapsed state (Figure 3c,d), a temperature cycle 50–25–50 °C was also carried out to check their thermoresponsive properties in the adsorbed wet state, and to compare the wet masses of the microgel films in the collapsed and swollen states (Figure 3c,d, steps 1′ and 1′′). Then, we compared the uptake of ChO by the P(NIPAM-*co*-APMA) and P(NIPAM-*co*-DMAPMA) microgel films, provided that, before the enzyme binding, they were in the swollen or in the collapsed state, depending on the temperature condition (Figure 3, step 2). Hence, we examined both a simple enzyme uptake by the microgel films and an enzyme uptake taking place with simultaneous microgel swelling, as already described for another microgel-enzyme system, that is, P(NIPAM-*co*-DMAPMA)/Tyr [10]. The adsorption of the microgel particles and the further incorporation of ChO into the deposited microgel film results in a decrease of *f* and an increase of *D* with spreading overtones (Figure 3), which denotes that the formed films exhibit viscoelastic properties. Afterwards, the subsequent modeling of *f* and *D*, according to the viscoelastic model by Voigt [35], correctly evaluates the adsorbed mass of the soft films (Table 1) along with other viscoelastic properties such as the layer viscosity and shear modulus (data not shown).

Comparing the data presented in Figure 3, one can clearly see that both the adsorption of the P(NIPAM-*co*-DMAPMA) microgel and the following uptake of ChO are very quick processes (Figure 3b,d). Furthermore, Table 1 shows that a considerably higher amount of this microgel is adsorbed at the elevated temperature. The latter finding is in agreement with the results of EKA, showing a more pronounced change in the surface charge of the MnO_2_-modified graphite-based surfaces after the deposition of the P(NIPAM-*co*-DMAPMA) microgel at 50 °C (Figure 2c). As follows from Figure 3d and Table 1, this temperature-induced increase in the amount of the adsorbed microgel, in turn, results in the considerable increase in the amount of ChO subsequently taken up by the microgel film. The similar behavior was already observed for the previously studied P(NIPAM-*co*-DMAPMA)/Tyr construct [10].

In the case of the P(NIPAM-*co*-APMA) microgel, however, we do not see such a strong temperature effect on the wet mass increase of the microgel film (Table 1). It appears that the temperature response is also more sluggish in the case of this microgel (Figure 3c, step 1′–1′′). The second clear difference is a notably slow enzyme uptake by the film of the P(NIPAM-*co*-APMA) microgel (Figure 3a,c, Step 2). One can see that the wet mass of this microgel deposited at 25 °C was comparable to that of the P(NIPAM-*co*-DMAPMA) one under the same adsorption conditions (Table 1). One would expect an even more pronounced uptake of ChO by the P(NIPAM-*co*-APMA) microgel film compared to a film of the P(NIPAM-*co*-DMAPMA) one, because of the higher content of chargeable amino groups in the P(NIPAM-*co*-APMA) microgel particles, and therefore, their potentially higher capacity. However, this is not observed. Indeed, a rather slow binding of ChO is found and the resultant mass of the enzyme, which was loaded, is lower compared to that observed in the case of the ChO taken up by the P(NIPAM-*co*-DMAPMA) microgel film deposited under the same conditions. The slow binding of the enzyme does not become considerably faster, even when the swelling film of the collapsed P(NIPAM-*co*-APMA) microgel, which was deposited at the elevated temperature, takes up ChO (Figure 3c, Step 2).

This experimental result allows us to suggest that the compared P(NIPAM-*co*-APMA) and P(NIPAM-*co*-DMAPMA) microgels have a different architecture. While most of the tertiary amino groups of the P(NIPAM-*co*-DMAPMA) microgel appear to be easily available for interacting with ChO, a certain fraction of the primary amino groups of the P(NIPAM-*co*-APMA) microgel may be hidden deeply in its denser inner part, being therefore considerably less accessible for the enzyme. It is also possible that the surface of the P(NIPAM-*co*-APMA) microgel resembles more the one of a pure PNIPAM microgel. This could be responsible for differences in the adhesive properties of the two microgels considered in this work, and manifests itself by the changes of their temperature-dependent adsorptive behavior.

### 3.3. Sensor Responses of Microgel/Enzyme Films

**Figure 4:**
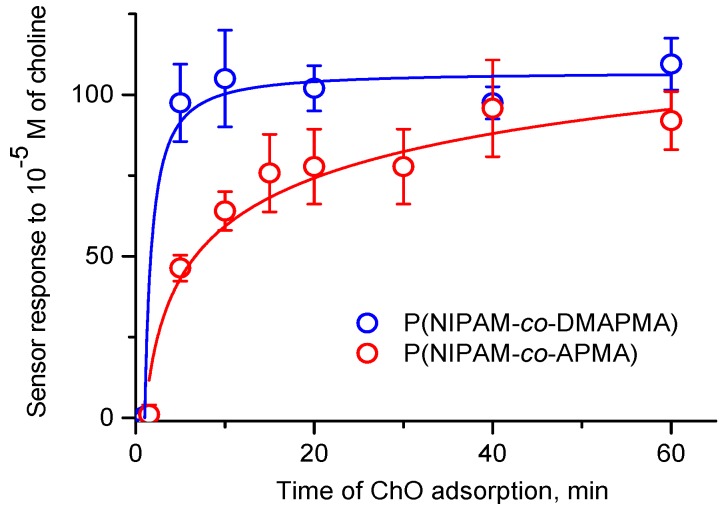
Sensor responses to choline (10^−5^ M) measured in 50 mM HEPES/30 mM KCl buffer (pH 7.5) at room temperature for the P(NIPAM-*co*-APMA)/ChO or P(NIPAM-*co*-DMAPMA)/ChO films vs. the enzyme adsorption time used for the biosensor preparation. Conditions of the fabrication of the microgel/enzyme films: (1) Adsorption of the microgels onto the SPE/MnO_2_-surface from 1 g/L solution at pH 9.3 at 50 °C for 1 h, followed by washing. (2) Uptake of ChO from 4 g/L solution at pH 7.0 at room temperature for a specified time, followed by washing. Lines through the experimentally obtained datapoints are drawn only as a guide to the eye. SPE stands for a screen-printed electrode.

To construct the biosensor surfaces, we fabricated microgel/enzyme films on the SPE/MnO_2_-surface, as follows. Firstly, the P(NIPAM-*co*-APMA) or P(NIPAM-*co*-DMAPMA) microgel was adsorbed at the specified temperature (25 or 50 °C), following with washing and drying cycles. Then, the ChO was allowed to interact with the films of the adsorbed microgels at an ambient temperature, following with the washing and drying of the resultant microgel/enzyme films. The sensor responses to choline for the P(NIPAM-*co*-APMA)/ChO or P(NIPAM-*co*-DMAPMA)/ChO films were obtained, depending on the enzyme adsorption time used for the biosensor preparation. The kinetic curves of the enzyme uptake again show different features (Figure 4). A very fast uptake of ChO by the P(NIPAM-*co*-DMAPMA) microgel film is observed, approaching saturation within 10 min. At the same time, the uptake of ChO by the P(NIPAM-*co*-APMA) microgel film appears to be a slower process, with the saturation being reached only within >40 min. The resultant sensor responses, which are clearly different for the P(NIPAM-*co*-APMA)/ChO and P(NIPAM-*co*-DMAPMA)/ChO films, again suggest architectural difference between the used microgels.

When comparing the microgel/enzyme films prepared under different adsorption conditions (Table 2), we also found a pronounced increase in the sensor responses obtained for the SPE/MnO_2_/P(NIPAM-*co*-DMAPMA)/ChO construct upon elevating the temperature of the deposition of the P(NIPAM-*co*-DMAPMA) microgel. These findings are in line with our former results [8,10]. Alternatively, there is only a slight increase in the sensor responses for the SPE/MnO_2_/P(NIPAM-*co*-APMA)/ChO construct with increasing the temperature of the deposition of the P(NIPAM-*co*-APMA) microgel. These inferences are, as a whole, similar to those we have made on the basis of the obtained QCM-D results, although some minor discrepancies happen to be because of different solid supports and adsorption conditions used. The increased time of the enzyme uptake (45 min), however, allow us to incorporate more ChO into the P(NIPAM-*co*-APMA) microgel film and come to a 1.5-2-fold increase in the corresponding sensor response. At the same time, the longer incubation times make the biosensor fabrication process time-consuming and lead to an increased scattering of the experimental datapoints.

It is worth noting that a non-homogeneous structure is well-known for PNIPAM-based microgels [36,37]. First of all, it results from a different density of the cross-links in the microgels having a denser inner part (core) and a looser outer (periphery) part (shell). Furthermore, the monomer units of a copolymerized ionic comonomer can be non-uniformly distributed throughout a microgel, being dependent on copolymerization constants. Apparently, the used P(NIPAM-*co*-APMA) microgel has rather high content of hardly accessible ionic comonomer units in the denser inner part (core), while the outer (periphery) part (shell) is hypothesized to be depleted of the APMA-moieties compared to the microgel’s core. This specific feature of the P(NIPAM-*co*-APMA) microgel restricts, to certain extent, the exploitation of the microgel’s thermosensitive behavior for the efficient modification of the surfaces and subsequent loading with enzymes.

Nevertheless, the P(NIPAM-*co*-APMA) microgel can still be considered as an advantageous polymeric candidate of a high capacity for the fabrication of the above biosensor constructs, although the strategy of the surface modification with this microgel should be altered. For example, a simple drop-casting at room temperature of the P(NIPAM-*co*-APMA) microgel dispersion onto the SPE/MnO_2_-surface, followed by the uptake of ChO by the resultant microgel film, allows us to prepare rather active biosensor constructs with the measured responses of 80 ± 8 nA (the enzyme adsorption for 45 min) and 58 ± 11 nA (the enzyme adsorption for 10 min).

Moreover, the presence of primary amino groups in the P(NIPAM-*co*-APMA) microgel allows us to stabilize the biosensor coating and to improve the sensor operational stability via enzyme-to-microgel cross-linking with GA. The operational stability, being very important parameter of biosensor performance, was evaluated for >10 repeated measurements at a fixed choline concentration (Supplementary Materials, Figure S4). It can be quantitatively described as a percentual change of the sensor response per measurement and calculated according to the Equation (2), as follows:

∆ = (tg*I*)/*I*_1_ × 100% (2)

where tg*I* is the slope of the dependence of the sensor response on the number of measurements normalized to the initial sensor response *I*_1_ and given in percent. According to this quantification, the closer the ∆-value reaches to zero, the higher the stability of the observed sensor response is. Table 3 demonstrates that the treatment of the SPE/MnO_2_/P(NIPAM-*co*-APMA)/ChO construct with a 1% (*v*/*v*) aqueous solution of GA for 1 h leads to significant improvement in the operational stability of the biosensor. It worth noting that the same treatment of the SPE/MnO_2_/P(NIPAM-*co*-DMAPMA)/ChO construct with GA does not lead to any stabilization of the biosensor, as the P(NIPAM-*co*-DMAPMA) microgel has tertiary amino groups, which are unable to enzyme-to-microgel cross-linking via GA, while the enzyme-to-enzyme cross-linking is prevented by a rarity of ionic moieties acting as binding sites for ChO. 

Thus, we demonstrate that the presence of primary amino groups in the microgel is very advantageous, from the point of view of a covalent immobilization of small biomolecules, like ChO. Moreover, the high operational stability of the biosensor constructs, obtained via treatment with GA, remarkably prolongs the time of practical application, thereby enabling precision assays in real samples coming, for example, from the environment or medicine.

## 4. Conclusions

In this work, we highlight the adsorptive behavior of the P(NIPAM-*co*-APMA) microgel, having primary amino groups, and the specific character of the subsequent loading of the adsorbed microgel with the small enzyme, ChO. We compared the results obtained for the P(NIPAM-*co*-APMA) microgel with those for the P(NIPAM-*co*-DMAPMA) one, which we applied in our previous studies. We demonstrate that a variation of the stimuli-dependent conditions during the adsorption of microgels controls their affinity toward the hydrophobic surface, thereby determining the efficiency of the surface modification, although the distribution of charged moieties within a microgel also appears to play a crucial role. We further show that the subsequent enzyme uptake by the adsorbed microgels and, in turn, the responses of the fabricated biosensor constructs, depend on the amount of the microgel deposited at the previous stage and can be governed by its state (swollen vs. collapsed). The architecture of the microgel should also be taken into account, and the enzyme uptake needs to be carefully examined and optimized individually for each microgel/enzyme pair. Finally, we highlight that the cross-linking of the enzyme to the microgel, the P(NIPAM-*co*-APMA) one, within such microgel/enzyme films considerably improves the operational stability of the fabricated biosensor constructs.

At this stage, we hypothesize that the microgel architecture, and the amount and chemical nature of functionalities, such as the ionic moieties introduced into the microgel in course of the copolymerization, all play important roles in the considered biosensor surface fabrication. To clarify this point, further studies are highly required, where these parameters will be varied in a systematic manner.

We also suppose that the reported covalent immobilization of enzymes into capacious microgels used in our work will allow for the design of promising smart nanosized adaptive systems with stimuli-controlled enzymatic activity. A macroscopic example of such a system was described by us previously for the PNIPAM macrogel containing covalently immobilized enzymes, alkaline phosphatase, and α-chymotrypsin [38,39,40].

## Figures and Tables

**Table 1:** Modeled masses of the microgel and microgel/enzyme films at various conditions of their fabrication.

	P(NIPAM-*co*-APMA)		P(NIPAM-*co*-DMAPMA)	
Temperature of the Microgel Adsorption	Mass of the Microgel Film, ng/cm^2^	Mass of the Enzyme Film, ng/cm^2^	Mass of the Microgel Film, ng/cm^2^	Mass of the enzyme Film, ng/cm^2^
25 °C	3960 ± 380	1840 ± 620	4770 ± 510	3510 ± 1300
50 °C	2310 ± 830 ^1^ 5080 ± 650 ^2^	5290 ± 1440	6410 ± 1360 ^1^ 10610 ± 1640 ^2^	15780 ± 1100

**^1^** Mass of the microgel film in its collapsed state (at 50 °C); ^2^ mass of the microgel film in its swollen state (at 25 °C); conditions: the microgels were adsorbed from 1 g/L solution at pH 9.3 at the specified temperature (25 or 50 °C); ChO was allowed to adsorb from 0.4 g/L solution at pH 7.0 at 25 °C, provided that the microgel film is either in its swollen or collapsed state. The data are given as the mean for the two experiments. P(NIPAM-*co*-DMAPMA) stands for poly(*N*-isopropylacrylamide-*co*-*N*-[3-(dimethylamino)propyl]methacrylamide); P(NIPAM-*co*-APMA) stands for poly(*N*-isopropyl acrylamide-*co*-*N*-(3-aminopropyl)methacrylamide).

**Table 2:** Sensor responses for different microgel/enzyme biosensor constructs.

Temperature of the Microgel Adsorption	Time of the Enzyme Uptake	Sensor Response to 10^−5^ M of Choline, nA	
		P(NIPAM-*co*-APMA)/ChO	P(NIPAM-*co*-DMAPMA)/ChO
25 °C	10 min	36 ± 7	29 ± 3
50 °C	10 min	46 ± 11	100 ± 13
	45 min	85 ± 34	–

**Table 3:** Comparative operational stability of different biosensor constructs.

Biosensor Construct	Treatment ^1^	Operational Stability, ∆, %
SPE/MnO_2_/P(NIPAM-*co*-APMA)/ChO	+ GA	−0.16 ± 0.13
	− GA	−3.53 ± 1.12
SPE/MnO_2_/P(NIPAM-*co*-DMAPMA)/ChO	+ GA	−4.20 ± 0.79
	− GA	−4.21 ± 0.78

^1^ + Glutar aldehyde (GA) means a treatment of the resultant surface with 1% aqueous GA solution for 1 h; − GA means a treatment of the reference surface with water for 1 h. SPE stands for a screen-printed electrode.

## Supplementary Materials

The following are available online at http://www.mdpi.com/2073-4360/10/7/791/s1, Figure S1: The temperature dependence of the hydrodynamic radius of P(NIPAM-*co*-DMAPMA) microgel particles; Figure S2: The pH-dependences of the hydrodynamic radius and ζ-potential of P(NIPAM-*co*-DMAPMA) microgel particles; Figure S3: The pH-dependence of the surface ζ-potential for the PVC film as measured by EKA; Figure S4: The relative biosensor responses of the SPE/MnO_2_/P(NIPAM-*co*-APMA)/ChO construct (open circles) and the SPE/MnO_2_/P(NIPAM-*co*-APMA)/ChO construct cross-linked with GA (solid circles) to consecutive additions of a choline solution with the concentration of 5 × 10^−5^ M.
