# Supplementary material for: Surface Functionalization by Stimuli-Sensitive Microgels for Effective Enzyme Uptake and Rational Design of Biosensor Setups
Source: Polymers (Basel). 2018 Jul 19;10(7):791. doi: 10.3390/polym10070791 (PMC6403641; doi:10.3390/polym10070791)
Supplement: Supplementary file 1 [file polymers-10-00791-s001.pdf]

polymers

Supplementary

Larisa V. Sigolaeva [1,*], Dmitry V. Pergushov [1], Marina Oelmann [2], Simona Schwarz [2], Monia Brugnoni [3], Ilya N. Kurochkin [1,4], Felix A. Plamper [3], Andreas Fery [2,5] and Walter Richtering [3]

1 Department of Chemistry, M.V. Lomonosov Moscow State University, Leninskie Gory 1/3, 119991 Moscow, Russia; lsigolaeva@belozersky.msu.ru (L.V.S); pergush@belozersky.msu.ru (D.V.P); ikur@genebee.msu.ru (I.N.K.)
2 Leibniz-Institut für Polymerforschung Dresden e.V., Hohe Str. 6, 01069 Dresden, Germany; oelmann@ipfdd.de (M.O.); simsch@ipfdd.de (S.S.); fery@ipfdd.de (A.F.)
3 Institute of Physical Chemistry II, RWTH Aachen University, Landoltweg 2, 52056 Aachen, Germany; brugnoni@pc.rwth-aachen.de (M.B.); plamper@pc.rwth-aachen.de (F.A.P.); richtering@rwth-aachen.de (W.R.)
4 N.M. Emanuel Institute of Biochemical Physics Russian Academy of Sciences, Kosygina Str. 4, Moscow, 119334 Russia
5 Technical University of Dresden, Physical Chemistry of Polymeric Materials, Hohe Str. 6, 01069 Dresden, Germany
* Correspondence: lsigolaeva@belozersky.msu.ru; Tel.: +7-495-939-40-42

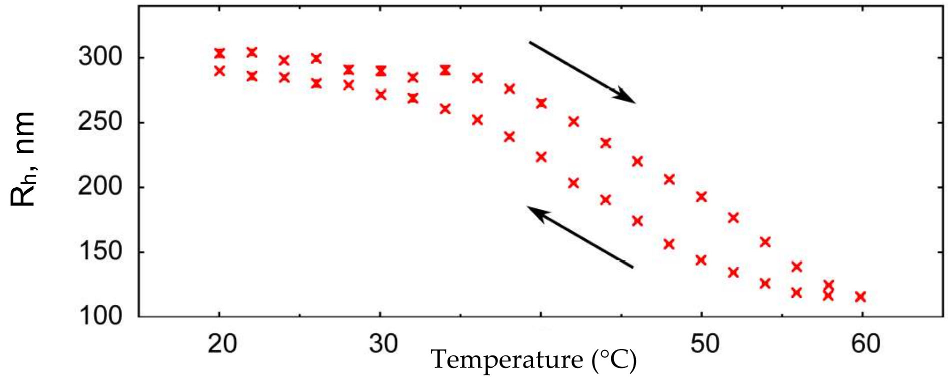

Figure S1. The temperature dependence of the hydrodynamic radius of poly(*N*-isopropylacrylamide-*co*-*N*-[3-(dimethylamino)propyl]methacrylamide) (P[NIPAM-*co*-DMAPMA]) microgel particles.

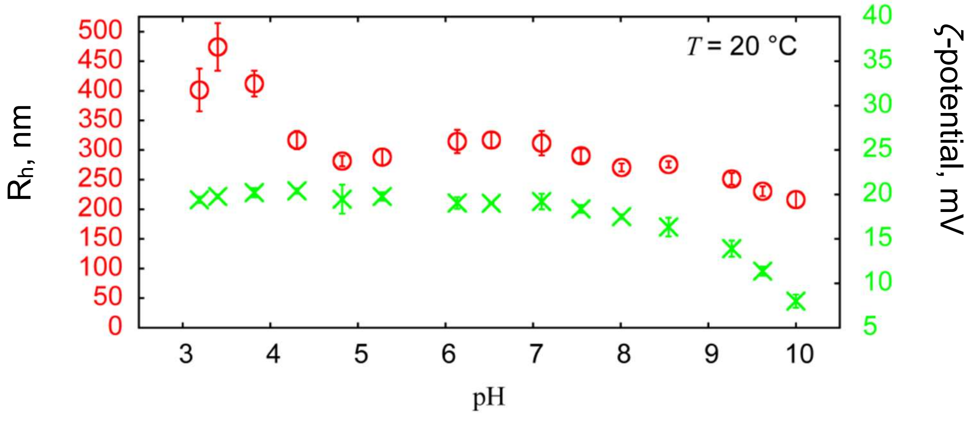

Figure S2. The pH-dependences of the hydrodynamic radius and ζ-potential of P(NIPAM-*co*-DMAPMA) microgel particles.

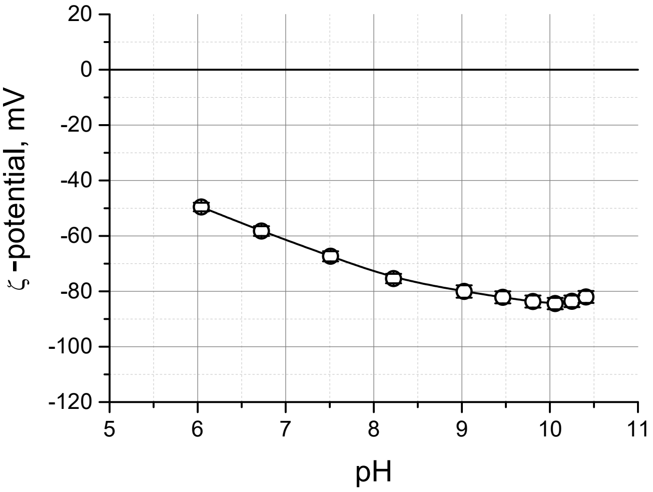

Figure S3. The pH-dependence of the surface ζ-potential of the poly(vinyl chloride) (PVC) film as measured by electrokinetic analysis (EKA).

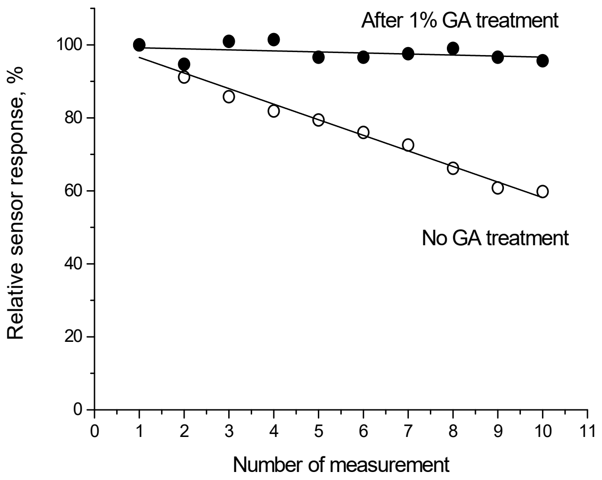

Figure S4. The relative biosensor responses of the screen-printed electrodes (SPE)/MnO_2/P(NIPAM-*co*-APMA)/ChO construct (open circles) and the SPE/MnO_2/P(NIPAM-*co*-APMA)/ChO construct cross-linked with glutar aldehyde (GA) (solid circles) to consecutive additions of choline solution, with the concentration of 5×10^{-5} M. The operational stability was characterized quantitatively as a percentage of change of the biosensor response per a single measurement, and was calculated according to the formula: $\Delta I = 100\% \times tgI/I_1$, where tg$I$ is the slope of the dependence of the biosensor response on the number of measurements, normalized to the initial biosensor response I_1 and given in percent.
